# Insight into pressure effect on optoelectronic, mechanical, and lattice vibrational properties of nanostructured Ga_x_In_1 − x_P_y_Sb_z_As_1 − y − z_ for the solar cells system

**DOI:** 10.1038/s41598-023-30681-1

**Published:** 2023-03-08

**Authors:** E. B. Elkenany, O. A. Alfrnwani, M. Sallah

**Affiliations:** 1 Physics Department, Faculty of Science, Mansoura University, Mansoura, 35516 Egypt; 2 Higher Institute of Engineering and Technology, New Damietta, 34517 Egypt

**Keywords:** Materials science, Nanoscience and technology, Physics

## Abstract

The electronic, optical, and elastic characteristics of the Ga_x_In_1 − x_P_y_Sb_z_As_1 − y − z_ alloy lattice matched to the GaSb substrate using a pseudo-potential formalism (EPM) based on the virtual crystal approximation (VCA) are performed. The mechanical features, acoustic velocity, and phonon frequencies of the Ga_x_In_1 − x_P_y_Sb_z_As_1 − y − z_/GaSb system are calculated. The sensitivity of these properties to pressure is considered. Our findings are reasonably consistent with the experimental evidence that is currently available. The studied properties of this alloy under the influence of pressure are a new achievement. Novel device applications would be made possible by the pentanary Ga_x_In_1 − x_P_y_Sb_z_As_1 − y − z_ alloy under high pressure.

## Introduction

The III-V quaternary semiconductor alloys are attractive options for a variety of device applications. Recent significant technical advancements have made it possible to create new devices, and the Groups III–V atoms are of tremendous technological significance in the creation of various optoelectronic devices^1^. The Ga_x_In_1 − x_P_y_Sb_z_As_1 − y − z_ alloy would enable novel device applications due to the formation of several wide spectrum ranges by correctly altering the composition component^2,3^. The energy band gap is well-known to be one of the best significant device parameters because it is strongly related to the operating wavelength of optoelectronic devices. Therefore, the ability to compute the energy band structure of semiconductor alloys is an essential requirement for any study of the phenomena occurring in these alloys as well as in the associated devices^4^.

The calculations presented here were performed using the empirical pseudo-potential technique (EPM)^5–10^. An averaged atomic separation and an averaged periodic potential are both employed in the virtual-crystal approximation (VCA)^11–13^. The optoelectronic features of the semiconductor alloys are important in the strategy and construction of devices. Knowing the optical-electronic energy levels, refractive indices, and optical dielectric constants of the material as a function of composition and wavelength is necessary for the design and study of these devices^1^. While much research has been done and is currently being done on the electronic and optical characteristics of semiconductors, the mechanical properties of semiconductors frequently place fundamental restrictions on the creation and manufacturing of current semiconductor devices^14–16^. In semiconductor physics, calculating the pressure dependence of the physical properties has long been a challenge. Recently, many studies have been performed on several semiconductor materials and alloys under the influence of pressure^17–21^.

This study presents a theoretical examination of the band parameters for Ga_x_In_1 − x_P_y_Sb_z_As_1 − y − z_ alloy lattice matched to GaSb substrate, based on the pseudo-potential approach. Quantities including energy gaps, mechanical parameters, sound velocity, and phonon frequencies are provided and compared with available experimental and prior theoretical data.

## Method and calculations

We employed an exclusive MATLAB-based code that was created specifically for our computations. Using the empirical pseudo-potential approach (EPM)^5,7–11^, the electronic energy spectra of Ga_x_In_1-x_P_y_Sb_z_As_1-y-z_ have been calculated. The pseudopotential formalism was used to assess the electron wave function. In the empirical pseudopotential technique (EPM), the atomic form factors are fitted to the experiment. It is a simple computation to solve for the band structure after the potential has been established. The following equation can be used to determine the relationship between the structural parameters x, y, and z from the lattice matching conditions of the pentanary alloy Ga_x_In_1 − x_P_y_Sb_z_As_1 − y − z_ on the GaSb substrate^22^:

1 $$y=\frac{{a}_{substrate}-0.033\mathrm{xz}+0.405\mathrm{x}-0.432\mathrm{z}-6.058}{-(0.013 x+0.189)}, 0<\mathrm{x}\le 1, 0<\mathrm{y}+\mathrm{z}\le 1$$

Where the lattice constant for GaSb is 6.096 Å. According to the virtual crystal approximation (VCA), the pseudopotential form factors for the Ga_x_In_1 − x_P_y_Sb_z_As_1 − y − z_ alloy under study are evaluated as follows:

2 $${W}_{alloy}^{S,A}(x,z,p)=xy{W}_{GaP}^{S,A}(p)+xz{W}_{GaSb}^{S,A}(p)+{x\left(1-y-z\right)W}_{GaAs}^{S,A}(p)+y\left(1-x\right){W}_{InP}^{S,A}(p)+z(1-x){W}_{InSb}^{S,A}(p)+{ (1-x)(1-y-z)W}_{InAs}^{S,A}(p)$$

The lattice constant of the pentanary alloy under investigation is obtained by using Vegard’s law ^23^

3 $${a}_{alloy}(x,z,p)=xy{a}_{GaP}(p)+xz{a}_{GaSb}(p)+{x(1-y-z)a}_{GaAs}(p)+y(1-x){a}_{InP}(p)+z(1-x){a}_{InSb}(p)+{(1-x)(1-y-z)a}_{InAs}(p)$$

The values of adjusted pseudopotential form factors and lattice constants are given in Table 1.

**Table 1:** The adjusted form factors for the alloy Ga_x_In_1-x_P_y_Sb_z_As_1-y_-z lattice matched to GaSb for pressures (0, 30, 60 kbar) at various values of composition.

a (Å)		p=0 kbar						p=30 kbar						p=60 kbar						p=90 kbar						p=120 kbar					
		6.0959						6.0002						5.9234						5.8593						5.8045					
z	x	w_s_^3^(a.u)	w_s_^8^(a.u)	w_s_^11^(a.u)	w_a_^3^(a.u)	w_a_^4^(a.u)	w_a_^11^(a.u)	w_s_^3^(a.u)	w_s_^8^(a.u)	w_s_^11^(a.u)	w_a_^3^(a.u)	w_a_^4^(a.u)	w_a_^11^(a.u)	w_s_^3^(a.u)	w_s_^8^(a.u)	w_s_^11^(a.u)	w_a_^3^(a.u)	w_a_^4^(a.u)	w_a_^11^(a.u)	w_s_^3^(a.u)	w_s_^8^(a.u)	w_s_^11^(a.u)	w_a_^3^(a.u)	w_a_^4^(a.u)	w_a_^11^(a.u)	w_s_^3^(a.u)	w_s_^8^(a.u)	w_s_^11^(a.u)	w_a_^3^(a.u)	w_a_^4^(a.u)	w_a_^11^(a.u)
0.2	0	− 0.1135	0.0099	0.0257	0.0339	0.0101	0.0271	− 0.1124	0.0104	0.0289	0.0348	0.0105	0.0275	− 0.1119	0.011	0.0328	0.0342	0.0109	0.0291	− 0.1109	0.0116	0.0365	0.0331	0.0112	0.03	− 0.1097	0.0117	0.04	0.0319	0.0114	0.0301
	0.1	− 0.1088	0.0054	0.0273	0.0302	0.0101	0.0248	− 0.1078	0.0057	0.0308	0.0307	0.0104	0.0251	− 0.1077	0.0061	0.0349	0.0293	0.0108	0.0266	− 0.1074	0.0065	0.0387	0.0278	0.011	0.0276	− 0.1071	0.0067	0.0422	0.026	0.0111	0.0279
0.4	0	− 0.124	0.0203	0.0185	0.0424	0.014	0.0215	− 0.1226	0.0214	0.0212	0.045	0.0148	0.0221	− 0.1212	0.0228	0.0254	0.0469	0.0153	0.0253	− 0.1185	0.024	0.0292	0.048	0.0161	0.027	− 0.1152	0.0243	0.0329	0.05	0.0166	0.0271
	0.1	− 0.1181	0.0149	0.0213	0.0378	0.0134	0.0201	− 0.1168	0.0158	0.0243	0.0398	0.0141	0.0206	− 0.1159	0.0169	0.0285	0.0407	0.0146	0.0235	− 0.1141	0.0179	0.0324	0.041	0.0152	0.0251	− 0.1118	0.0181	0.0361	0.042	0.0154	0.0251
	0.2	− 0.1131	0.0103	0.0232	0.0338	0.0131	0.0183	− 0.1119	0.0109	0.0265	0.0353	0.0136	0.0187	− 0.1114	0.0118	0.0308	0.0354	0.0141	0.0213	− 0.1103	0.0126	0.0347	0.0351	0.0145	0.0228	− 0.109	0.0128	0.0384	0.0353	0.0147	0.023
	0.3	− 0.1091	0.0063	0.0243	0.0303	0.0129	0.016	− 0.108	0.0067	0.0279	0.0313	0.0132	0.0165	− 0.1078	0.0075	0.0323	0.0308	0.0138	0.0188	− 0.1073	0.0082	0.0363	0.0302	0.0141	0.0203	− 0.1067	0.0085	0.04	0.0299	0.0143	0.0208
0.6	0.2	− 0.1211	0.0191	0.0183	0.0404	0.0158	0.0144	− 0.1197	0.0202	0.0211	0.0434	0.0167	0.0151	− 0.1185	0.0217	0.0254	0.0455	0.0172	0.0188	− 0.1161	0.0229	0.0294	0.0468	0.0179	0.0207	− 0.1131	0.0231	0.0332	0.0493	0.0181	0.0205
	0.3	− 0.1158	0.0143	0.0206	0.036	0.0151	0.0131	− 0.1146	0.0151	0.0236	0.0384	0.0158	0.0136	− 0.1138	0.0165	0.0279	0.0396	0.0163	0.0169	− 0.1121	0.0174	0.0319	0.0404	0.0167	0.0187	− 0.1101	0.0176	0.0357	0.0419	0.0169	0.0185
	0.4	− 0.1114	0.0102	0.022	0.0321	0.0145	0.0114	− 0.1104	0.0108	0.0253	0.0339	0.0151	0.0119	− 0.1099	0.0119	0.0296	0.0345	0.0155	0.0147	− 0.1089	0.0128	0.0337	0.0348	0.0159	0.0163	− 0.1076	0.013	0.0374	0.0358	0.0161	0.0164
	0.5	− 0.1079	0.0066	0.0226	0.0287	0.0142	0.0093	− 0.107	0.0071	0.0262	0.0301	0.0145	0.0098	− 0.1068	0.0081	0.0306	0.0301	0.015	0.0122	− 0.1062	0.0089	0.0346	0.0302	0.0153	0.0137	− 0.1056	0.0092	0.0385	0.0307	0.0156	0.0142
0.8	0.4	− 0.1169	0.0174	0.0194	0.0369	0.0161	0.0093	− 0.1158	0.0184	0.0221	0.04	0.0171	0.0098	− 0.1148	0.02	0.0263	0.042	0.0174	0.0135	− 0.1128	0.021	0.0303	0.0435	0.0178	0.0151	− 0.1103	0.021	0.034	0.0459	0.0178	0.0144
	0.5	− 0.1122	0.0131	0.0211	0.0326	0.0152	0.0081	− 0.1112	0.0139	0.0241	0.0351	0.0159	0.0085	− 0.1106	0.0152	0.0282	0.0364	0.0162	0.0116	− 0.1093	0.0161	0.0322	0.0373	0.0165	0.013	− 0.1077	0.0162	0.0359	0.039	0.0165	0.0124
	0.6	− 0.1083	0.0094	0.0221	0.0288	0.0144	0.0065	− 0.1076	0.01	0.0254	0.0307	0.0149	0.0069	− 0.1072	0.0112	0.0294	0.0314	0.0153	0.0094	− 0.1064	0.012	0.0333	0.0321	0.0155	0.0106	− 0.1054	0.0121	0.0371	0.0332	0.0155	0.0102
	0.7	− 0.1053	0.0064	0.0222	0.0255	0.0138	0.0046	− 0.1048	0.0068	0.0259	0.0269	0.0141	0.005	− 0.1046	0.0078	0.0299	0.0272	0.0145	0.0068	− 0.1042	0.0086	0.0338	0.0277	0.0147	0.0079	− 0.1036	0.0088	0.0377	0.0285	0.0148	0.008
1	0.6	− 0.1113	0.0151	0.0217	0.0318	0.0148	0.0062	− 0.1106	0.0159	0.0244	0.0348	0.0158	0.0065	− 0.11	0.0175	0.028	0.0365	0.0158	0.0093	− 0.1086	0.0182	0.0318	0.0377	0.0159	0.0103	− 0.1068	0.018	0.0354	0.0397	0.0156	0.0086
	0.7	− 0.1071	0.0113	0.023	0.0276	0.0137	0.0051	− 0.1067	0.0119	0.026	0.0299	0.0144	0.0053	− 0.1063	0.0132	0.0295	0.031	0.0144	0.0074	− 0.1055	0.0138	0.0332	0.0318	0.0144	0.008	− 0.1044	0.0136	0.0368	0.0331	0.0141	0.0066
	0.8	− 0.1037	0.0081	0.0236	0.0239	0.0126	0.0038	− 0.1035	0.0085	0.0268	0.0256	0.0132	0.0038	− 0.1034	0.0096	0.0302	0.0262	0.0132	0.0052	− 0.103	0.0101	0.0339	0.0268	0.0132	0.0056	− 0.1024	0.01	0.0375	0.0276	0.0129	0.0045
	0.9	− 0.1012	0.0054	0.0234	0.0206	0.0118	0.0021	− 0.1011	0.0057	0.027	0.0218	0.0121	0.0021	− 0.1011	0.0066	0.0303	0.022	0.0122	0.0027	− 0.101	0.0071	0.0339	0.0225	0.0122	0.0029	− 0.1007	0.0071	0.0377	0.0231	0.012	0.0023
	1	− 0.0995	0.0034	0.0224	0.0178	0.0111	0	− 0.0995	0.0034	0.0264	0.0184	0.0112	0	− 0.0995	0.0042	0.0298	0.0184	0.0113	0	− 0.0995	0.0048	0.0333	0.019	0.0114	0	− 0.0995	0.0049	0.0373	0.0195	0.0114	0

The elastic constants C_11_, C_12_, and C_44_ for the investigated alloy can be determined as in Refs.^24–28^. We utilized the crystal density (g) and the stiffness constants (C_ij_) to determine the sound velocity as follows^29^:

4 $$\mathrm{v}=\sqrt{\frac{{\mathrm{c}}_{\mathrm{ij}}}{\mathrm{g}}}$$

The bulk (B_u_), Young’s (Y_0_), shear moduli (C_s_), and other important mechanical quantities can be determined as in Ref.^30^. The longitudinal and transverse phonon frequencies (LO and TO) were calculated using the Lyddane-Sachs-Teller relations^31,32^

5 $$\frac{{\upomega }_{\mathrm{TO}}^{2}}{{\upomega }_{\mathrm{LO}}^{2}}=\frac{{\upvarepsilon }_{\infty }}{{\upvarepsilon }_{\mathrm{o}}}$$

6 $${\upomega }_{\mathrm{LO}}^{2}-{\upomega }_{\mathrm{TO}}^{2}=\frac{4\uppi {{\mathrm{e}}_{\mathrm{T}}^{*}}^{2}{\mathrm{e}}^{2}}{\mathrm{M}{\Omega }_{\mathrm{o}}{\upvarepsilon }_{\infty }}$$

where ε_0_, ε_∞_, Ω, M, e, e_T_* are the static dielectric constant, high-frequency dielectric constant, volume occupied by one atom, twice the reduced mass, electron charge, and transverse effective charge, respectively.

## Results and discussion

**Figure 1:**
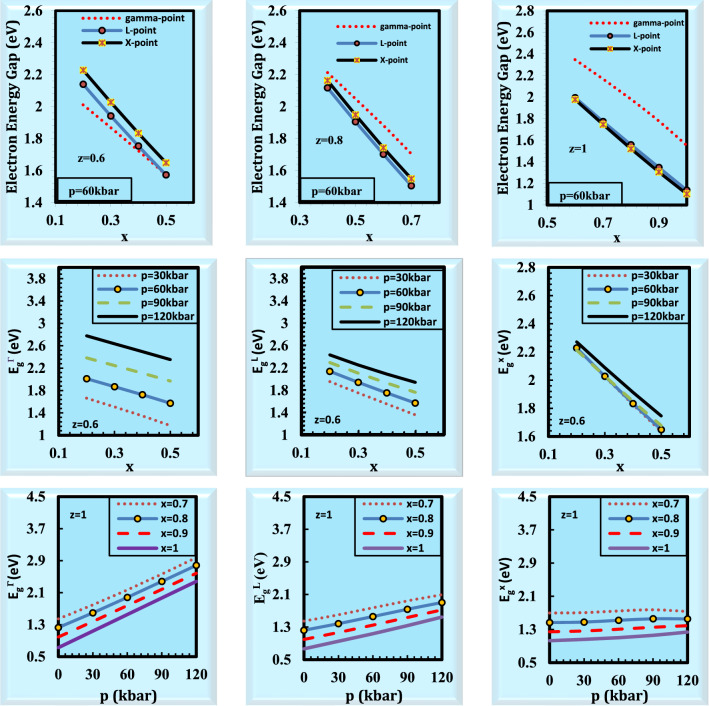
Energy band gaps (E_g_^L^, E_g_^Г^, E_g_^X^) versus composition and pressure in Ga_x_In_1 − x_P_y_Sb_z_As_1 − y − z_ lattice matched to GaSb.

**Figure 2:**
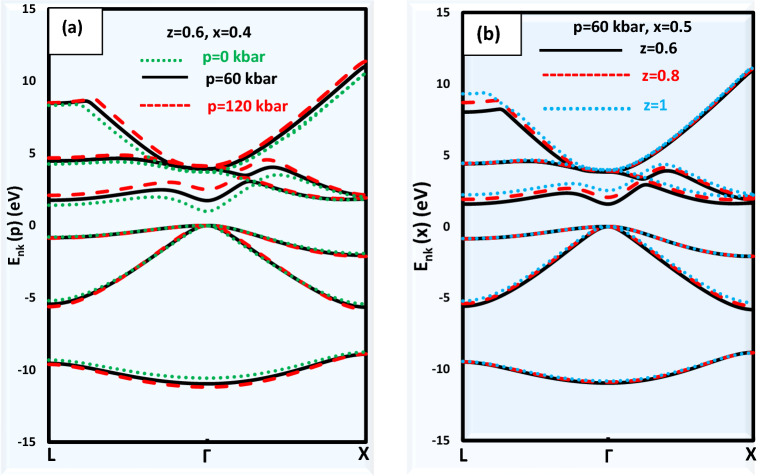
The energy band structure for Ga_x_In_1 − x_P_y_Sb_z_As_1 − y − z_ alloy lattice matched to GaSb substrate (**a**) as a function of pressure at z = 0.6, x = 0.4, and (**b**) as a function of composition at p = 60 kbar, x = 0.5.

The finest semiconductors for applications involving the emission of photons by radiative recombination of electrons and holes are direct band gap semiconductors^33^. In solid-state physics, the band structure is one of the essential principles. The significance of energy band theories for crystalline solids stems from the fact that energy band structure may be used to easily explain several significant physical and chemical characteristics. The energy band gaps for the alloy Ga_x_In_1-x_P_y_Sb_z_As_1-y-z_ lattice matched to GaSb for various values of pressure and compositions are listed in Table 2 and displayed in Fig. 1. The band structure changes with various pressure/compositions for Ga_x_In_1-x_P_y_Sb_z_As_1-y-z_ alloy lattice matched to GaSb substrate is displayed in Fig. 2. It is noted from Fig. 2a and b that the conduction bands are shifted upward under the influence of pressure and composition. It is seen that the conduction band is more affected by pressure and composition than the valence bands. The effect of pressure and composition is more pronounced in the first conduction band than in the other conduction bands. Therefore, the electrical features of the alloy under investigation could be easily calculated.

**Table 2:** The energy band gaps and the transition from direct to indirect semiconductor for the alloy Ga_x_In_1 − x_P_y_Sb_z_As_1 − y − z_ lattice matched to GaSb for various values of pressure and compositions.

z	x	p = 0 kbar			p = 30 kbar			p = 60 kbar			p = 90 kbar			p = 120 kbar			Transition from direct to indirect semiconductor
		E_g_^L^ (eV)	E_g_^Г^ (eV)	E_g_^X^ (eV)	E_g_^L^ (eV)	E_g_^Г^ (eV)	E_g_^X^ (eV)	E_g_^L^ (eV)	E_g_^Г^ (eV)	E_g_^X^ (eV)	E_g_^L^ (eV)	E_g_^Г^ (eV)	E_g_^X^ (eV)	E_g_^L^ (eV)	E_g_^Г^ (eV)	E_g_^X^ (eV)	
0.2	0.0	1.31	0.58	1.68	1.42	0.91	1.68	1.57	1.26	1.68	1.71	1.62	1.67	**1.84**	**1.97**	**1.67**	At p = 120 kbar, Г→X
	0.1	1.14	0.46	1.52	1.25	0.81	1.51	1.42	1.17	1.51	**1.58**	**1.53**	**1.52**	**1.73**	**1.89**	**1.53**	At p = 90, 120 kbar, Г→X
0.4	0.0	1.76	1.06	2.16	1.89	1.40	2.17	2.06	1.74	2.17	2.20	2.10	2.14	**2.32**	**2.47**	**2.16**	At p = 120 kbar, Г→X
	0.1	1.58	0.94	1.98	1.71	1.28	1.99	1.88	1.63	1.99	**2.03**	**1.99**	**1.97**	**2.16**	**2.36**	**2.00**	At p = 90, 120 kbar, Г→X
	0.2	1.4	0.8	1.8	1.53	1.15	1.81	1.71	1.51	1.81	**1.87**	**1.88**	**1.81**	**2.01**	**2.24**	**1.84**	At p = 90, 120 kbar, Г→X
	0.3	1.22	0.64	1.63	1.35	1.01	1.63	1.54	1.39	1.63	**1.72**	**1.76**	**1.65**	**1.88**	**2.13**	**1.69**	At p = 90, 120 kbar, Г→X
0.6	0.2	1.81	1.31	2.21	1.96	1.66	2.23	2.14	2.01	2.23	**2.30**	**2.38**	**2.21**	**2.43**	**2.78**	**2.27**	At p = 90, 120 kbar, Г→X
	0.3	1.61	1.15	2.01	1.75	1.51	2.03	1.94	1.87	2.03	**2.11**	**2.25**	**2.03**	**2.25**	**2.64**	**2.09**	At p = 90, 120 kbar, Г→X
	0.4	1.41	0.98	1.81	1.56	1.35	1.83	1.75	1.72	1.83	**1.93**	**2.11**	**1.85**	**2.09**	**2.50**	**1.91**	At p = 90, 120 kbar, Г→X
	0.5	1.21	0.79	1.63	1.36	1.17	1.64	1.57	1.57	1.65	**1.76**	**1.97**	**1.68**	**1.94**	**2.35**	**1.75**	At p = 90, 120 kbar, Г→X
0.8	0.4	1.77	1.5	2.14	1.93	1.86	2.17	**2.12**	**2.21**	**2.16**	**2.28**	**2.60**	**2.17**	**2.43**	**3.01**	**2.25**	At p = 60 kbar, Г→L
	0.5	1.56	1.31	1.92	1.71	1.68	1.94	**1.91**	**2.05**	**1.95**	2.08	2.44	1.97	2.23	2.85	2.05	At p = 60 kbar, Г→L
	0.6	1.34	1.11	1.71	1.50	1.49	1.73	**1.70**	**1.88**	**1.74**	1.89	2.28	1.78	2.06	2.68	1.86	At p = 60 kbar, Г→L
	0.7	1.13	0.9	1.5	1.29	1.29	1.52	**1.50**	**1.70**	**1.55**	1.70	2.12	1.60	1.89	2.52	1.68	At p = 60 kbar, Г→L
1	0.6	1.67	1.64	1.94	**1.82**	**1.99**	**1.95**	2.00	2.35	1.98	2.16	2.74	2.00	2.30	3.16	1.93	At p = 30 kbar, Г→L
	0.7	1.45	1.44	1.7	**1.60**	**1.80**	**1.71**	1.77	2.17	1.75	1.94	2.56	1.78	2.09	2.98	1.73	At p = 30 kbar, Г→L
	0.8	**1.22**	**1.23**	**1.47**	1.38	1.59	1.48	1.56	1.98	1.52	1.74	2.38	1.56	1.90	2.78	1.55	At p = 0 kbar, Г→L
	0.9	1	0.99	1.24	**1.17**	**1.38**	**1.26**	1.35	1.78	1.31	1.54	2.18	1.35	1.72	2.58	1.39	At p = 30 kbar, Г→L
	1	0.77, 0.78^a^	0.73, 0.72^a^	1.03, 1.01^a^	**0.95**	**1.14**	**1.06**	1.14	1.55	1.10	1.34	1.97	1.16	1.55	2.38	1.24	At p = 30 kbar, Г→L

^a^Ref.^35^.

For the fixed value of pressure (60 kbar), it is seen the direct and indirect energy band gaps at (Γ, L, X) points for z = 0.6, 0.8, and 1 are decreased by increasing composition x. For a certain value of z (0.6), the direct and indirect energy band gaps (E_g_^Γ^, E_g_^L^, and E_g_^X^) at different values of pressure (0, 30, 60, 90, and 120 kbar) reduce by raising the composition x. It is noted that at z = 1, the energy band gaps (E_g_^Γ^, and E_g_^L^) at various compositions x are enhanced by enhancing pressure, but E_g_^X^ changes irregularly as in Table 2. It is deduced that the alloy under investigation is a direct semiconductor at p = 0 kbar for all values of z and x except at z = 1, and x = 0.8 the alloy becomes an indirect semiconductor. The alloy under investigation is observed to be a direct semiconductor at p = 30 kbar up to z = 1, x = 0.6, at which point the alloy starts to convert into an indirect semiconductor. It can be observed that the alloy under study is a direct semiconductor at p = 60 kbar except for z = 0.8 and x = 0.4, the alloy begins to change into an indirect semiconductor after these values. For p = 90 kbar, the alloy under examination is a direct semiconductor at (z = 0.2, x = 0), and (z = 0.4, x = 0) except for these values, the alloy is an indirect semiconductor. It is also obvious that the alloy under consideration is an indirect semiconductor at p = 120 kbar for all values of z and x. The transition from direct to indirect semiconductor for each pressure and composition is displayed in Table 2. Semiconductor direct bandgap semiconductors are ideal if you need to absorb photons in a very tiny piece of semiconductor^34^. The calculated results at p = 0 kbar, z = 1, and x = 1 (y = 0, GaSb) give an excellent agreement with the published data^35^. The results at the rest values of pressure and composition are new and predicted. It is observed from Fig. 2a that the top of valence bands and the minimum of conduction bands are located at the same point (Γ-point) for p = 0 and 60 kbar. Therefore, this alloy at z = 0.6, and x = 0.4 for the pressure values (p = 0, 60 kbar) is a direct semiconductor material, but at 120 kbar is converted to an indirect one. Direct band gap semiconductors often have a smaller effective mass of electrons^36,37^. It is noticed from Fig. 2a that the energy band gap at Γ-point is increased by increasing pressure. To effectively absorb both low- and high-energy photons, solar cells employ semiconductors with progressively greater bandgaps as one moves deeper into the wafer^38^. The electrons move more quickly in direct bandgap semiconductor materials than they do in indirect bandgap semiconductor materials^36,37^. This challenge is highlighted by the development of multi-junction solar cells^39^. By selecting semiconductors with the proper band gaps, it is possible to reduce heat losses brought on by thermalization and therefore produce an efficient solar cell^40^. The most efficient solar energy conversion materials have band gap energies in the range of 1 to 1.8 eV when considering the energy distribution of the solar spectrum^40^. They are hence excellent for use with lasers and LEDs^41^.

**Figure 3:**
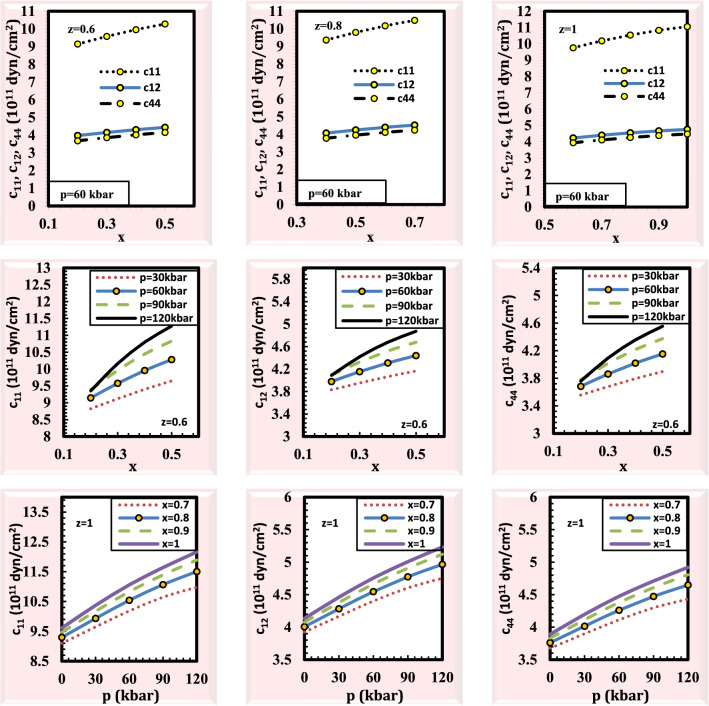
Elastic constants for the alloy Ga_x_In_1 − x_P_y_Sb_z_As_1 − y − z_ lattice matched to GaSb as a function of pressure and composition.

To better understand the mechanical stability of the material under study, the crystal elastic constants must be determined. The elastic constants can be used to determine how stable a crystal is across a wide range of pressure and composition. Elastic constants play a crucial role in describing the mechanical characteristics of materials. The independent elastic constants are used to calculate the origin of Poisson’s ratio, Debye temperature, and low thermal conductivity. The calculated results at p = 0 kbar, z = 1, x = 1 (y = 0) give the results for the binary material GaSb which are in excellent agreement with the experimental and theoretical data^42–45^ (Supplementary Table 3). This comparison gives support for the calculated results at the rest values of pressure. Figure 3 shows that the elastic constants C_11_, C_12_, and C_44_ for the alloy Ga_x_In_1-x_P_y_Sb_z_As_1-y-z_ lattice matched to GaSb as a function of pressure and compositions. It has been shown that at particular pressure values, increasing composition causes the elastic parameters C_11_, C_12_, and C_44_ to increase. Also, it is demonstrated that increasing pressure leads to enhancing the elastic parameters C_11_, C_12_, and C_44_ at specific values of composition. The conditions C_11_>0, C_44_>0, C_11_–C_12_>0, and C_11_+2C_12_>0^46,47^ are satisfied throughout wide pressure and composition ranges. The computed findings according to these conditions demonstrate the stability of the structure of Ga_x_In_1-x_P_y_Sb_z_As_1-y-z_ alloy.

**Figure 4:**
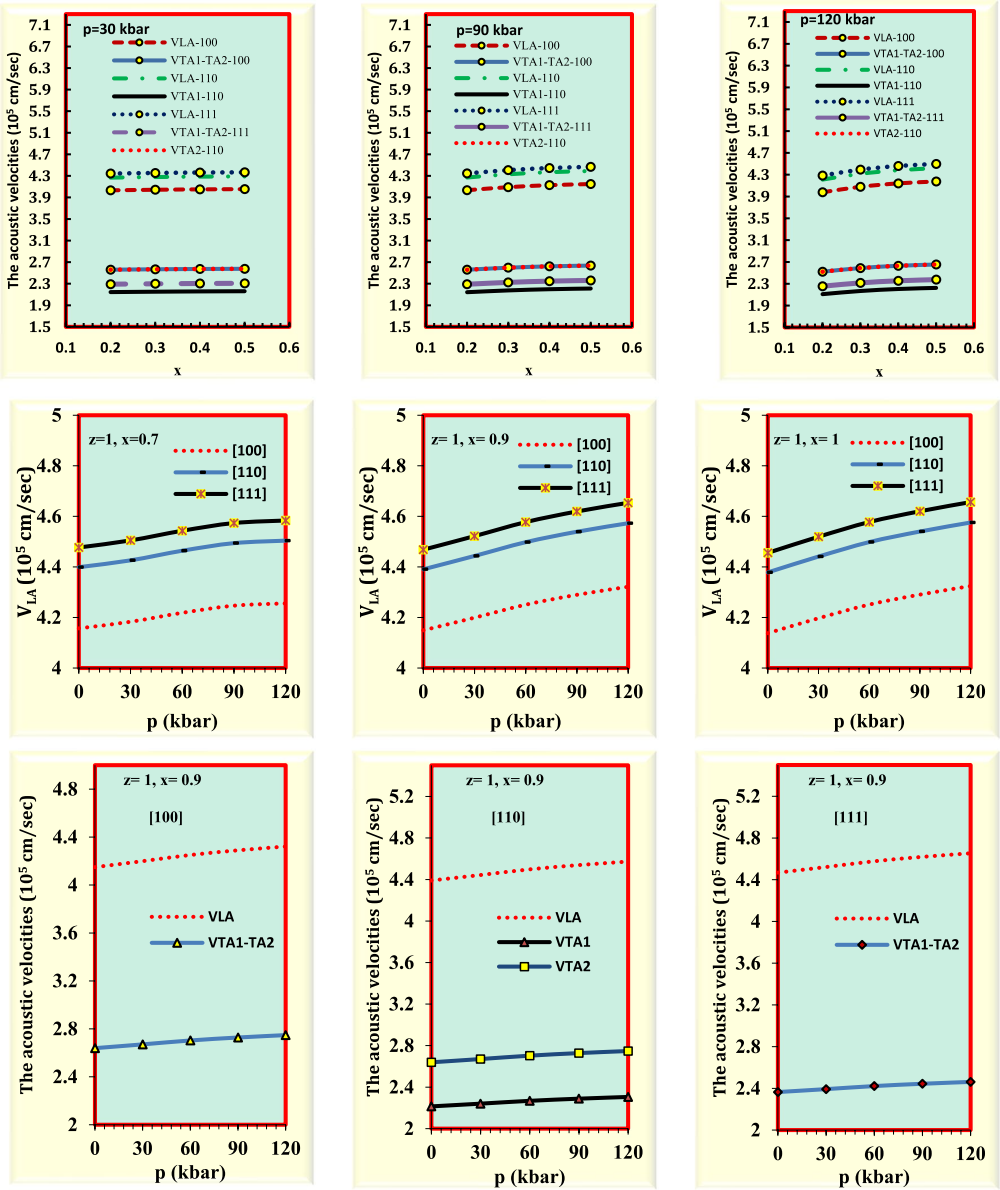
The acoustic velocities of Ga_x_In_1 − x_P_y_Sb_z_As_1 − y − z_ for GaSb substrate as a function of composition (x) and pressure (p).

The wave propagation through a medium is described by the acoustic wave velocity. In other words, the velocity at which a small disturbance will propagate through the medium is called acoustic velocity^48^. It is possible to proceed with the bulk sound velocity after determining the elastic constants. Figure 4 depicts how the acoustic wave velocity changes concerning the crystal orientation, composition, and pressure. The direction of propagation has a significant impact on the sound velocities in anisotropic solid materials. For a fixed value of pressure, the acoustic wave velocity is increased by enhancing composition. Also, for a certain value of composition, the sound velocity rises by raising pressure from 0 to 120 kbar. The agreement of calculated results with experimental and theoretical results is uniformly good for most of the sound velocity components^29,49^ (Supplementary Table 4). Due to the lack of both experimental and theoretical data regarding the sound velocity at high values of pressure for the considered alloy, our results are predictions and may serve as a reference for future works.

**Figure 5:**
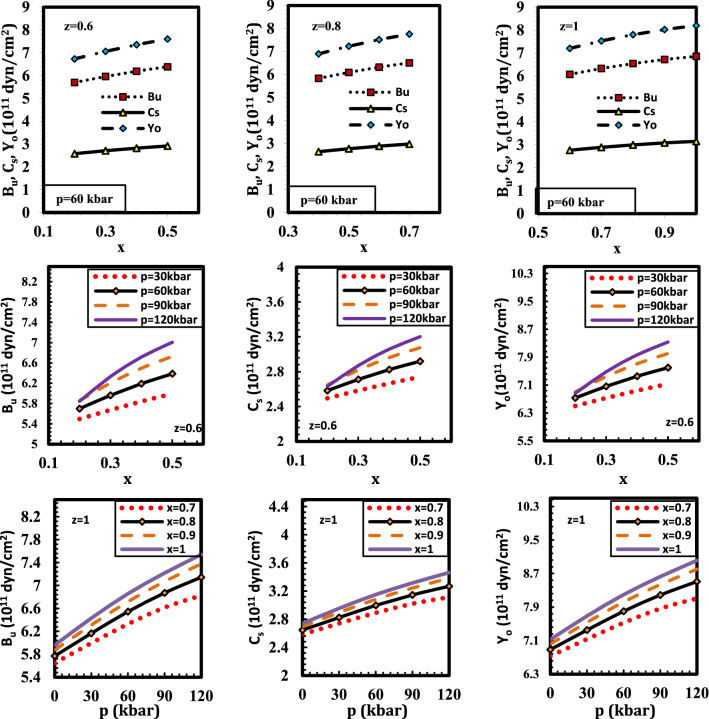
The variation of elastic moduli for the alloy Ga_x_In_1 − x_P_y_Sb_z_As_1 − y − z_ lattice matched to GaSb with pressure and composition.

The ductile-brittle nature of materials is often discussed in terms of the mechanical constants of the relevant material. By taking into account the bulk modulus B_u_ as the resistance to fracture and the shear modulus C_s_ as the resistance to plastic deformation, one may estimate the brittle and ductile behaviors of materials^50^. According to this formula, a material's ductile and brittle behavior may be distinguished by a B_u_/C_s_ ratio of around 1.75; i.e. If B_u_/C_s_ is more than 1.75, a material will behave ductility; if not, it will act brittlely. The ductility and brittleness of semiconductor alloys' mechanical characteristics are crucial for their applications. The calculated value of Young’s, shear, and bulk moduli at p=0 kbar, z=1, x=1 (y=0) agrees reasonably well with the experimental data^29,44,51^ (Supplementary Table 5). Figure 5 displays that the mechanical moduli for the alloy Ga_x_In_1 − x_P_y_Sb_z_As_1 − y − z_ lattice matched to GaSb as a function of pressure and composition. It is noted that Young’s, shear, and bulk moduli are enhanced by enhancing pressure and composition. The calculated B_u_/C_s_ ratios for Ga_x_In_1 − x_P_y_Sb_z_As_1 − y − z_ are more than 1.75. This means that the alloy under investigation is a ductile material over the whole region of pressure and composition. When the value of Young’s modulus is high, the material is stiff. The calculated Young’s modulus indicates that the Ga_x_In_1 − x_P_y_Sb_z_As_1 − y − z_ is stiff throughout the whole pressure and composition range.

**Figure 6:**
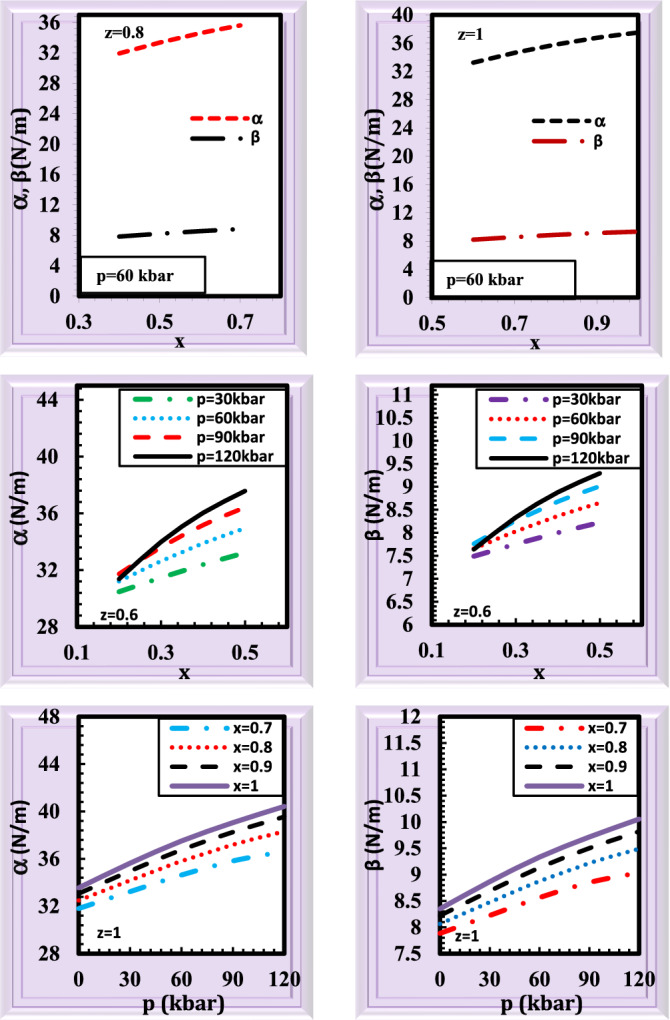
The bond-stretching (α), and bond-bending (β) force constants for the alloy Ga_x_In_1 − x_P_y_Sb_z_As_1 − y − z_ lattice matched to GaSb as a function of pressure and composition.

It is necessary to understand mechanical characteristics, particularly the elastic constants, which define how macroscopic stress reacts, to understand how strain affects electronic properties^52^. It has been crucial to study the bond-stretching and bond-bending force constants of semiconductors due to the possibility of using these semiconductors in many optoelectronic devices such as detectors, lasers, integrated circuits, modulators, light-emitting diodes, and filters^53^. Based on information acquired from lattice vibration data, the bond-stretching and bond-bending force constants are dependent on the nearest neighbor distance. The calculated bond-stretching (α), and bond-bending (β) force constants for the alloy Ga_x_In_1 − x_P_y_Sb_z_As_1 − y − z_ lattice matched to GaSb as a function of the Phosphorus concentration and pressure are displayed in Fig. 6. Once again a satisfactory agreement is observed between our calculated results and the experimental data concerning p = 0 kbar, z = 1, x = 1 (y = 0)^54,55^ (Supplementary Table 6). There are no measurements or ab initio calculations at high pressures of α, and β for Ga_x_In_1 − x_P_y_Sb_z_As_1 − y − z_ known to us. In view of Fig. 6, one can note that the bond-bending force constant increases with increasing composition and pressure. The same qualitative behavior could be seen for the variation of the bond-stretching force constant.

**Figure 7:**
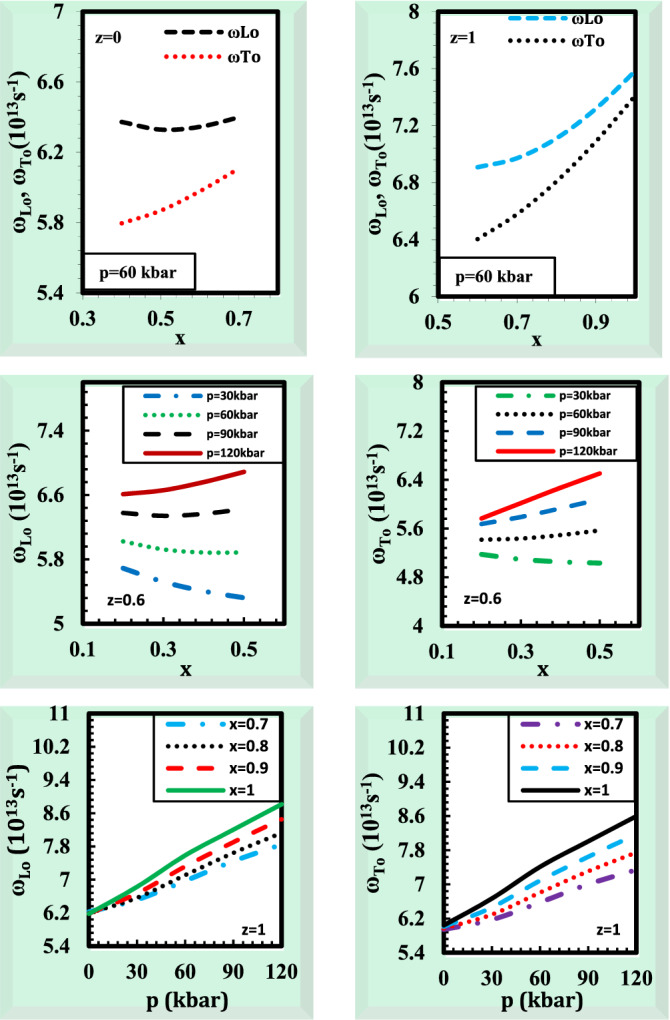
The variation of phonon frequencies ω_LO_ and ω_TO_ for the alloy Ga_x_In_1 − x_P_y_Sb_z_As_1 − y − z_ lattice matched to GaSb with pressure and composition.

It is important to determine the lattice vibrations properties under high-pressure circumstances. Due to its influence on material characteristics including thermal, electrical, and optical conductions. It is significant to give information on basic vibrational properties in the anionic and cationic alloys under study. In studying the optical and transport characteristics of materials, the longitudinal and transversal phonons are essential. The estimation of the phonon frequencies is very motivating because it provides some insight into the infrared activity of phonons in materials^56^. The Born effective charge of the ions is a key quantity in the analysis of the dynamics of crystalline lattices. This amount may be used to determine the ionicity and is implicitly connected to the bond polarity of the substance^42^. Figure 7 presents the effects of pressure and composition on the phonon frequencies ω_LO_ and ω_TO_ for the alloy Ga_x_In_1 − x_P_y_Sb_z_As_1 − y − z_ lattice matched to GaSb. Increasing the pressure causes the phonon frequencies ω_LO_ and ω_TO_ to increase as displayed in Fig. 7. This result is explained by the fact that a rise in pressure causes a rise in the force constants, which then causes a rise in the bulk modulus, which then causes a rise in the frequencies. Correspondingly, the phonon frequencies ω_LO_ and ω_TO_ are changed as the composition rises. This is due to the force constants being dependent on the composition and values of the lattice parameters. The results are compared with the experimental and theoretical data^22,42,49^ which are only available for binary compound GaSb at p = 0 kbar, z = 1, x = 1, y = 0 and showed generally satisfactory agreement^22,42,49^ (Supplementary Table 7). In the absence of the experimental values for pentanary alloys of interest at high values of pressure, our calculated values may serve as a reference.

## Conclusions

In this work, we calculated the electronic, optical, mechanical properties, acoustic velocity, and phonon frequencies of the Ga_x_In_1 − x_P_y_Sb_z_As_1 − y − z_ alloy lattice matched to the GaSb substrate by using an EPM with VCA under the influence of pressure and composition. The transitions from the direct to indirect band gap were identified at various pressure and composition values. It was determined that the material under examination was mechanically stable over a range of pressure and composition. The ductile-brittle nature of the considered alloy within a range of pressure and composition was discussed. The results showed that the phonon frequencies increase as the pressure increases. Our results mostly agree with the experimental data that are already available. Because there are no experimental or theoretical findings for the researched values for the relevant semiconductor alloy at high pressures, the estimated results in the current study are likely to be useful as a reference. The pentanary Ga_x_In_1 − x_P_y_Sb_z_As_1 − y − z_ alloy at high pressure could be used for novel device applications.

## Supplementary Information

Supplementary Information 1.

Supplementary Information 2.

Supplementary Information 3.

Supplementary Information 4.

Supplementary Information 5.

## Data Availability

The data that support the findings of this research are available from the corresponding author upon reasonable request.

## References

[1] Bouarissa N (2001). Optoelectronic properties of InAs1− xPx semiconducting alloys. Mater. Sci. Eng. B.

[2] Shim K, Rabitz H (1999). Electronic and structural properties of the pentanary alloy Ga x In 1–x P y Sb z As 1− y− z. J. Appl. Phys..

[3] Shim K, Rabitz H (1998). Independent and correlated composition behavior of material properties: Application to energy band gaps for the Ga α In 1− α P β As 1− β and Ga α In 1− α P β Sb γ As 1− β− γ alloys. Phys. Rev. B.

[4] Bouarissa N (1999). The effect of compositional disorder on electronic band structure in GaxIn1− xAsySb1− yalloys lattice matched to GaSb. Superlattices Microstruct..

[5] Degheidy AR, Elkenany EB, Alfrnwani OA (2018). Mechanical properties of AlPxSb1-x semiconductor alloys under the effect of temperature and pressure. Comput. Condens. Matter.

[6] Degheidy AR, Elkenany EB, Madkour MAK, AbuAli AM (2018). Temperature dependence of phonons and related crystal properties in InAs, InP and InSb zinc-blende binary compounds. Comput. Condens. Matter.

[7] Cahn RN, Cohen ML (1970). Local pseudopotential model for GaSb: Electronic and optical properties. Phys. Rev. B.

[8] Chelikowsky JR, Cohen ML (1976). Nonlocal pseudopotential calculations for the electronic structure of eleven diamond and zinc-blende semiconductors. Phys. Rev. B.

[9] Elkenany EB (2016). Optoelectronic and mechanical properties of InSb semiconductor under the effect of temperature. Silicon.

[10] Degheidy AR, Elkenany EB (2011). Effect of pressure and temperature on electronic structure of GaN in the zinc-blende structure. Semiconductors.

[11] Degheidy AR, Elkenany EB (2013). Effect of temperature and pressure on the electronic structure of Ga xIn1-xAsyP1-y alloys lattice matched to GaAs substrate. Mater. Chem. Phys..

[12] Degheidy AR, Elkenany EB (2016). Pressure and composition dependence of electronic, optical and mechanical properties of GaPxSb1 - X alloys. Thin Solid Films.

[13] Degheidy AR, Elkenany EB (2012). The response of temperature and hydrostatic pressure of zinc-blende Ga xIn1-xAs semiconducting alloys. Chin. Phys. B.

[14] Degheidy AR, Elkenany EB (2015). Theoretical studies of optoelectronic and mechanical properties of GaPxSb1-x alloys under the effect of temperature. Mater. Chem. Phys..

[15] Faber KT, Malloy KJ (1992). The Mechanical Properties of Semiconductors.

[16] Degheidy AR, Elkenany EB, Alfrnwani OA (2018). Influence of composition, temperature and pressure on the optoelectronic and mechanical properties of InPxSb1-x alloys. Comput. Condens. Matter.

[17] Degheidy AR, Elkenany EB (2013). Electronic and optical properties of InAs1-xPx alloys under the effect of temperature and pressure. Thin Solid Films.

[18] Zeng X (2018). Pressure effect on elastic constants and related properties of Ti3Al intermetallic compound: A first-principles study. Materials (Basel).

[19] Başer P, Elagoz S (2017). The hydrostatic pressure and temperature effects on hydrogenic impurity binding energies in lattice matched InP/In0. 53Ga0. 47As/InP square quantum well. Superlattices Microstruct..

[20] Othman MS (2020). Mechanical response of PbSSe, PbSTe ternary and PbSnSTe quaternary alloys at high pressure. ARO-THE Sci. J. KOYA Univ..

[21] Degheidy AR, Elkenany EB, Alfrnwani O (2018). Mechanical properties of AlxIn1-xSb ternary alloys under the effect of pressure and temperature. Comput. Condens. Matter.

[22] Baaziz H, Charifi Z, Bouarissa N (2006). Dynamical effective charges and dielectric constants in the pentanary alloy GaxIn1− xPySbzAs1− y− z lattice matched to InAs and GaSb. Mater. Lett..

[23] Vegard L (1921). Die konstitution der mischkristalle und die raumfüllung der atome. Zeitschrift für Phys..

[24] Bouarissa N (2006). Elastic constants and acoustical phonon properties of GaAsxSb1− x. Mater. Chem. Phys..

[25] Jamal M, Asadabadi SJ, Ahmad I, Aliabad HAR (2014). Elastic constants of cubic crystals. Comput. Mater. Sci..

[26] Ferahtia S, Saib S, Bouarissa N (2019). Computational studies of mono-chalcogenides ZnS and ZnSe at high-pressures. Results Phys..

[27] Daoud S, Loucif K, Bioud N, Lebgaa N, Belagraa L (2012). Effect of hydrostatic pressure on the structural, elastic and electronic properties of (B3) boron phosphide. Pramana.

[28] Baranowski JM (1984). Bond lengths, force constants and local impurity distortions in semiconductors. J. Phys. C.

[29] Adachi S (2005). Properties of Group-iv, III-v and II-VI Semiconductors.

[30] Algarni H, Al-Hagan OA, Bouarissa N, Alhuwaymel TF, Khan MA (2018). Elastic constants and mechanical stability of In x Al1− x As y Sb1− y lattice-matched to different substrates. Philos. Mag..

[31] Davydov SY, Tikhonov SK (1998). Pressure dependence of the dielectric and optical properties of wide-gap semiconductors. Semiconductors.

[32] Kittel C, McEuen P, McEuen P (1976). Introduction to Solid State Physics.

[33] Queisser HJ (2009). Detailed balance limit for solar cell efficiency. Mater. Sci. Eng. B.

[34] Valenti M, Jonsson MP, Biskos G, Schmidt-Ott A, Smith WA (2016). Plasmonic nanoparticle-semiconductor composites for efficient solar water splitting. J. Mater. Chem. A.

[35] Bouarissa N (2003). Theoretical study of electronic and positronic properties in Ga x ln 1–x P y Sb z As 1-y-z lattice matched to GaSb. Eur. Phys. J. B.

[36] Tsymbalov E (2021). Machine learning for deep elastic strain engineering of semiconductor electronic band structure and effective mass. npj Comput. Mater..

[37] Goodman CHL (1982). Direct-gap group IV semiconductors based on tin. IEE Proc. I (Solid-State Electron Devices).

[38] Bagher AM, Vahid MMA, Mohsen M (2015). Types of solar cells and application. Am. J. Opt. Photonics.

[39] Avrutin V, Izyumskaya N, Morkoç H (2011). Semiconductor solar cells: Recent progress in terrestrial applications. Superlattices Microstruct..

[40] Paranthaman MP, Wong-Ng W, Bhattacharya RN (2016). Semiconductor Materials for Solar Photovoltaic Cells.

[41] Chow WW, Koch SW (1999). Semiconductor-Laser Fundamentals: Physics of the Gain Materials.

[42] Baaziz H, Charifi Z, Bouarissa N (2001). Ionicity and transverse effective charge in GaxIn1− xAsySb1− y quaternary alloy semiconductors. Mater. Chem. Phys..

[43] Bouarissa N (2003). Compositional dependence of the elastic constants and the Poisson ratio of GaxIn1− xSb. Mater. Sci. Eng. B.

[44] Elkenany EB (2015). Theoretical investigations of electronic, optical and mechanical properties for GaSb and AlSb semiconductors under the influence of temperature. Spectrochim. Acta A Mol. Biomol. Spectrosc..

[45] Cardona M, Peter YY (2005). Fundamentals of Semiconductors.

[46] Zhang X, Ying C, Li Z, Shi G (2012). First-principles calculations of structural stability, elastic, dynamical and thermodynamic properties of SiGe, SiSn. GeSn. Superlattices Microstruct..

[47] Degheidy AR, Alfrnwani OA, Elkenany EB (2021). Thermal and pressure dependence of mechanical properties for AlxIn1−xPySb1−y/GaSb system. Bull. Mater. Sci..

[48] Everest FA, Pohlmann KC (2022). Master Handbook of Acoustics.

[49] Elkenany EB (2021). Acoustic velocity and phonon frequencies of GaxIn1-xSb alloys under pressure, temperature, and compositions. Phys. Scr..

[50] Pugh SF (1954). XCII. Relations between the elastic moduli and the plastic properties of polycrystalline pure metals. London Edinburgh Dublin Philos. Mag. J. Sci..

[51] Levinshtein M (1997). Handbook Series on Semiconductor Parameters.

[52] Bouarissa N, Kassali K (2001). Mechanical properties and elastic constants of Zinc-Blende Ga1—xInxN Alloys. Phys. Status Solidi.

[53] Verma AS (2008). Bond-stretching and bond-bending force constant of binary tetrahedral (AIIIBV and AIIBVI) semiconductors. Phys. Lett. A.

[54] Harrison WA, Ciraci S (1974). Bond-orbital model. II. Phys. Rev. B.

[55] Zerroug S, Sahraoui FA, Bouarissa N (2006). Elastic properties of AlxIn1− xPySb1− y and AlxGa1− xPySb1− y lattice matched to InAs substrate. Mater. Lett..

[56] Bouarissa N (2007). Phonon frequencies and related parameters in GaxIn1− xSb and InAsxP1− x. Phys. B Condens. Matter.

