# Supplementary material for: Insight into pressure effect on optoelectronic, mechanical, and lattice vibrational properties of nanostructured GaxIn1 − xPySbzAs1 − y − z for the solar cells system
Source: Sci Rep. 2023 Mar 8;13:3891. doi: 10.1038/s41598-023-30681-1 (PMC9995325; doi:10.1038/s41598-023-30681-1)
Supplement: Supplementary file 1 — Supplementary Information 1. [file 41598_2023_30681_MOESM1_ESM.docx]

| **Table 3.** The polarity and elastic constants in (Dyn/cm^2^) for the alloy Ga_x_In_1-x_P_y_Sb_z_As_1-y-z_ lattice matched to GaSb for various values of pressure and compositions. | | | | | | | | | | | | | | | | | | | | | |
| --- | --- | --- | --- | --- | --- | --- | --- | --- | --- | --- | --- | --- | --- | --- | --- | --- | --- | --- | --- | --- | --- |
|  |  | p= 0 kbar | | | | p=30 kbar | | | | p= 60 kbar | | | | p= 90 kbar | | | | p=120 kbar | | | |
| z | x | α_p_ | C_11_ | C_12_ | C_44_ | α_p_ | C_11_ | C_12_ | C_44_ | α_p_ | C_11_ | C_12_ | C_44_ | α_p_ | C_11_ | C_12_ | C_44_ | α_p_ | C_11_ | C_12_ | C_44_ |
| 0.2 | 0.0 | 0.3 | 8.76 | 3.79 | 3.54 | 0.31 | 9.38 | 4.06 | 3.79 | 0.31 | 10.05 | 4.34 | 4.06 | 0.30 | 10.68 | 4.62 | 4.31 | 0.29 | 11.28 | 4.87 | 4.55 |
|  | 0.1 | 0.28 | 8.94 | 3.86 | 3.61 | 0.28 | 9.62 | 4.15 | 3.88 | 0.27 | 10.37 | 4.48 | 4.19 | 0.26 | 11.08 | 4.78 | 4.48 | 0.24 | 11.77 | 5.07 | 4.76 |
| 0.4 | 0.0 | 0.34 | 8.36 | 3.62 | 3.37 | 0.37 | 8.77 | 3.81 | 3.54 | 0.39 | 9.10 | 3.96 | 3.67 | 0.40 | 9.37 | 4.08 | 3.77 | 0.43 | 9.38 | 4.10 | 3.77 |
|  | 0.1 | 0.32 | 8.57 | 3.71 | 3.46 | 0.34 | 9.06 | 3.93 | 3.65 | 0.35 | 9.54 | 4.14 | 3.85 | 0.36 | 9.97 | 4.33 | 4.02 | 0.38 | 10.24 | 4.45 | 4.12 |
|  | 0.2 | 0.3 | 8.76 | 3.79 | 3.54 | 0.32 | 9.32 | 4.03 | 3.76 | 0.32 | 9.92 | 4.29 | 4.00 | 0.32 | 10.46 | 4.53 | 4.22 | 0.32 | 10.90 | 4.72 | 4.40 |
|  | 0.3 | 0.28 | 8.94 | 3.86 | 3.61 | 0.29 | 9.57 | 4.13 | 3.86 | 0.29 | 10.24 | 4.43 | 4.14 | 0.28 | 10.86 | 4.69 | 4.39 | 0.28 | 11.40 | 4.92 | 4.60 |
| 0.6 | 0.2 | 0.33 | 8.44 | 3.65 | 3.4 | 0.36 | 8.82 | 3.83 | 3.56 | 0.38 | 9.14 | 3.98 | 3.68 | 0.40 | 9.39 | 4.09 | 3.78 | 0.44 | 9.35 | 4.09 | 3.76 |
|  | 0.3 | 0.31 | 8.65 | 3.74 | 3.49 | 0.33 | 9.12 | 3.95 | 3.68 | 0.35 | 9.58 | 4.15 | 3.86 | 0.36 | 9.97 | 4.33 | 4.02 | 0.38 | 10.16 | 4.42 | 4.09 |
|  | 0.4 | 0.29 | 8.85 | 3.82 | 3.58 | 0.31 | 9.40 | 4.07 | 3.79 | 0.31 | 9.96 | 4.31 | 4.02 | 0.32 | 10.45 | 4.52 | 4.22 | 0.33 | 10.80 | 4.68 | 4.36 |
|  | 0.5 | 0.27 | 9.03 | 3.9 | 3.65 | 0.28 | 9.65 | 4.17 | 3.90 | 0.28 | 10.28 | 4.44 | 4.15 | 0.28 | 10.83 | 4.68 | 4.37 | 0.29 | 11.28 | 4.87 | 4.55 |
| 0.8 | 0.4 | 0.32 | 8.61 | 3.72 | 3.47 | 0.35 | 9.01 | 3.91 | 3.63 | 0.37 | 9.36 | 4.07 | 3.77 | 0.39 | 9.64 | 4.19 | 3.88 | 0.42 | 9.65 | 4.21 | 3.88 |
|  | 0.5 | 0.29 | 8.83 | 3.82 | 3.57 | 0.32 | 9.32 | 4.03 | 3.76 | 0.33 | 9.79 | 4.24 | 3.95 | 0.34 | 10.19 | 4.42 | 4.11 | 0.36 | 10.41 | 4.52 | 4.20 |
|  | 0.6 | 0.27 | 9.03 | 3.9 | 3.65 | 0.29 | 9.60 | 4.15 | 3.88 | 0.29 | 10.17 | 4.39 | 4.11 | 0.30 | 10.65 | 4.60 | 4.30 | 0.32 | 11.00 | 4.76 | 4.44 |
|  | 0.7 | 0.24 | 9.21 | 3.97 | 3.72 | 0.26 | 9.86 | 4.25 | 3.98 | 0.26 | 10.48 | 4.52 | 4.24 | 0.27 | 11.02 | 4.75 | 4.45 | 0.28 | 11.45 | 4.94 | 4.62 |
| 1 | 0.6 | 0.29 | 8.87 | 3.83 | 3.58 | 0.31 | 9.33 | 4.04 | 3.77 | 0.33 | 9.76 | 4.23 | 3.94 | 0.35 | 10.12 | 4.39 | 4.08 | 0.37 | 10.29 | 4.47 | 4.15 |
|  | 0.7 | 0.26 | 9.1 | 3.92 | 3.68 | 0.28 | 9.65 | 4.17 | 3.90 | 0.29 | 10.19 | 4.40 | 4.11 | 0.30 | 10.65 | 4.60 | 4.30 | 0.32 | 10.98 | 4.75 | 4.43 |
|  | 0.8 | 0.23 | 9.3 | 4.01 | 3.76 | 0.25 | 9.93 | 4.28 | 4.01 | 0.25 | 10.54 | 4.55 | 4.26 | 0.26 | 11.07 | 4.77 | 4.47 | 0.27 | 11.50 | 4.97 | 4.65 |
|  | 0.9 | 0.2 | 9.47 | 4.08 | 3.83 | 0.22 | 10.17 | 4.38 | 4.11 | 0.22 | 10.83 | 4.66 | 4.38 | 0.22 | 11.39 | 4.91 | 4.61 | 0.23 | 11.89 | 5.12 | 4.81 |
|  | 1 | 0.18,  0.24^a^ | 9.61,  9.08^b^ | 4.13,  3.91^b^ | 3.89,  3.82^c^,  3.65^d^ | 0.18 | 10.37 | 4.46 | 4.20 | 0.19 | 11.06 | 4.76 | 4.47 | 0.19 | 11.64 | 5.01 | 4.71 | 0.20 | 12.16 | 5.23 | 4.92 |
| ^a^Ref. ^41^, ^b^Ref.^42^,^c^Ref.^43^, ^d^Ref.^44^. | | | | | | | | | | | | | | | | | | | | | |
