# Supplementary material for: Insight into pressure effect on optoelectronic, mechanical, and lattice vibrational properties of nanostructured GaxIn1 − xPySbzAs1 − y − z for the solar cells system
Source: Sci Rep. 2023 Mar 8;13:3891. doi: 10.1038/s41598-023-30681-1 (PMC9995325; doi:10.1038/s41598-023-30681-1)
Supplement: Supplementary file 2 — Supplementary Information 2. [file 41598_2023_30681_MOESM2_ESM.docx]

| **Table 4.** Sound speed in (10^5^ cm/s) propagating along the three major directions [100], [110], and [111] for the alloy Ga_x_In_1-x_P_y_Sb_z_As_1-y-z_ lattice matched to GaSb for various values of pressure and compositions. | | | | | | | | | | | | | | | | | | | | | | | | | | | | | | | |
| --- | --- | --- | --- | --- | --- | --- | --- | --- | --- | --- | --- | --- | --- | --- | --- | --- | --- | --- | --- | --- | --- | --- | --- | --- | --- | --- | --- | --- | --- | --- | --- |
|  |  | p= 0 kbar | | | | | | p= 30 kbar | | | | | | p= 60 kbar | | | | | | p= 90 kbar | | | | | | p= 120 kbar | | | | | |
|  |  | [100] | | [110] | | [111] | | [100] | | [110] | | [111] | | [100] | | [110] | | [111] | | [100] | | [110] | | [111] | | [100] | | [110] | | [111] | |
| z | x | V_LA_ | V_TA1-TA2_ | V_LA_ | V_TA1_ | V_LA_ | V_TA1-TA2_ | V_LA_ | V_TA1-TA2_ | V_LA_ | V_TA1_ | V_LA_ | V_TA1-TA2_ | V_LA_ | V_TA1-TA2_ | V_LA_ | V_TA1_ | V_LA_ | V_TA1-TA2_ | V_LA_ | V_TA1-TA2_ | V_LA_ | V_TA1_ | V_LA_ | V_TA1-TA2_ | V_LA_ | V_TA1-TA2_ | V_LA_ | V_TA1_ | V_LA_ | V_TA1-TA2_ |
| 0.2 | 0.0 | 3.98 | 2.53 | 4.22 | 2.12 | 4.29 | 2.27 | 4.03 | 2.56 | 4.27 | 2.15 | 4.34 | 2.29 | 4.10 | 2.60 | 4.34 | 2.18 | 4.41 | 2.33 | 4.16 | 2.65 | 4.41 | 2.22 | 4.48 | 2.37 | 4.22 | 2.68 | 4.47 | 2.25 | 4.55 | 2.40 |
|  | 0.1 | 3.97 | 2.53 | 4.2 | 2.12 | 4.28 | 2.26 | 4.03 | 2.56 | 4.26 | 2.15 | 4.34 | 2.29 | 4.10 | 2.61 | 4.34 | 2.19 | 4.42 | 2.34 | 4.17 | 2.65 | 4.42 | 2.23 | 4.50 | 2.38 | 4.24 | 2.70 | 4.49 | 2.26 | 4.57 | 2.42 |
| 0.4 | 0.0 | 4 | 2.54 | 4.23 | 2.13 | 4.31 | 2.27 | 4.02 | 2.55 | 4.25 | 2.14 | 4.33 | 2.28 | 4.03 | 2.56 | 4.26 | 2.14 | 4.34 | 2.29 | 4.03 | 2.56 | 4.27 | 2.14 | 4.34 | 2.29 | 3.99 | 2.53 | 4.22 | 2.12 | 4.30 | 2.26 |
|  | 0.1 | 4 | 2.54 | 4.23 | 2.13 | 4.3 | 2.27 | 4.02 | 2.55 | 4.26 | 2.14 | 4.33 | 2.29 | 4.06 | 2.58 | 4.29 | 2.16 | 4.37 | 2.31 | 4.09 | 2.60 | 4.33 | 2.17 | 4.40 | 2.32 | 4.09 | 2.60 | 4.33 | 2.17 | 4.41 | 2.32 |
|  | 0.2 | 3.99 | 2.54 | 4.22 | 2.13 | 4.3 | 2.27 | 4.02 | 2.56 | 4.26 | 2.14 | 4.33 | 2.29 | 4.08 | 2.59 | 4.31 | 2.17 | 4.39 | 2.32 | 4.12 | 2.62 | 4.36 | 2.20 | 4.44 | 2.35 | 4.15 | 2.64 | 4.40 | 2.21 | 4.47 | 2.36 |
|  | 0.3 | 3.98 | 2.53 | 4.21 | 2.12 | 4.29 | 2.27 | 4.02 | 2.56 | 4.26 | 2.14 | 4.33 | 2.29 | 4.09 | 2.60 | 4.32 | 2.18 | 4.40 | 2.33 | 4.14 | 2.63 | 4.38 | 2.21 | 4.46 | 2.36 | 4.19 | 2.66 | 4.43 | 2.23 | 4.51 | 2.38 |
| 0.6 | 0.2 | 4.03 | 2.56 | 4.26 | 2.14 | 4.34 | 2.29 | 4.03 | 2.56 | 4.27 | 2.14 | 4.34 | 2.29 | 4.04 | 2.56 | 4.27 | 2.15 | 4.35 | 2.29 | 4.03 | 2.56 | 4.27 | 2.14 | 4.35 | 2.29 | 3.98 | 2.52 | 4.21 | 2.11 | 4.29 | 2.26 |
|  | 0.3 | 4.02 | 2.56 | 4.26 | 2.14 | 4.33 | 2.29 | 4.04 | 2.57 | 4.28 | 2.15 | 4.35 | 2.30 | 4.07 | 2.58 | 4.31 | 2.17 | 4.38 | 2.31 | 4.09 | 2.60 | 4.33 | 2.18 | 4.41 | 2.32 | 4.08 | 2.59 | 4.32 | 2.17 | 4.39 | 2.32 |
|  | 0.4 | 4.02 | 2.56 | 4.26 | 2.14 | 4.33 | 2.29 | 4.05 | 2.57 | 4.29 | 2.16 | 4.36 | 2.30 | 4.09 | 2.60 | 4.33 | 2.18 | 4.41 | 2.33 | 4.13 | 2.62 | 4.37 | 2.20 | 4.45 | 2.35 | 4.14 | 2.63 | 4.38 | 2.20 | 4.46 | 2.36 |
|  | 0.5 | 4.02 | 2.55 | 4.25 | 2.14 | 4.32 | 2.29 | 4.05 | 2.58 | 4.29 | 2.16 | 4.37 | 2.31 | 4.11 | 2.61 | 4.35 | 2.19 | 4.42 | 2.34 | 4.15 | 2.64 | 4.39 | 2.21 | 4.47 | 2.36 | 4.18 | 2.65 | 4.42 | 2.22 | 4.50 | 2.38 |
| 0.8 | 0.4 | 4.08 | 2.59 | 4.31 | 2.17 | 4.39 | 2.32 | 4.08 | 2.59 | 4.32 | 2.17 | 4.40 | 2.32 | 4.09 | 2.60 | 4.33 | 2.17 | 4.40 | 2.32 | 4.09 | 2.60 | 4.33 | 2.17 | 4.41 | 2.32 | 4.04 | 2.56 | 4.28 | 2.15 | 4.35 | 2.29 |
|  | 0.5 | 4.08 | 2.59 | 4.32 | 2.17 | 4.39 | 2.32 | 4.10 | 2.60 | 4.34 | 2.18 | 4.41 | 2.33 | 4.12 | 2.62 | 4.36 | 2.20 | 4.44 | 2.35 | 4.14 | 2.63 | 4.39 | 2.21 | 4.46 | 2.36 | 4.13 | 2.62 | 4.38 | 2.20 | 4.45 | 2.35 |
|  | 0.6 | 4.08 | 2.59 | 4.31 | 2.17 | 4.39 | 2.32 | 4.11 | 2.61 | 4.35 | 2.19 | 4.42 | 2.34 | 4.15 | 2.64 | 4.39 | 2.21 | 4.47 | 2.36 | 4.18 | 2.66 | 4.42 | 2.23 | 4.50 | 2.38 | 4.19 | 2.66 | 4.44 | 2.23 | 4.51 | 2.38 |
|  | 0.7 | 4.07 | 2.59 | 4.31 | 2.17 | 4.39 | 2.32 | 4.11 | 2.61 | 4.35 | 2.19 | 4.43 | 2.34 | 4.16 | 2.65 | 4.40 | 2.22 | 4.48 | 2.37 | 4.20 | 2.67 | 4.44 | 2.24 | 4.52 | 2.39 | 4.22 | 2.68 | 4.47 | 2.25 | 4.55 | 2.40 |
| 1 | 0.6 | 4.15 | 2.64 | 4.4 | 2.21 | 4.47 | 2.36 | 4.17 | 2.65 | 4.41 | 2.22 | 4.49 | 2.37 | 4.18 | 2.66 | 4.43 | 2.23 | 4.51 | 2.38 | 4.20 | 2.67 | 4.44 | 2.23 | 4.52 | 2.39 | 4.18 | 2.65 | 4.42 | 2.22 | 4.50 | 2.37 |
|  | 0.7 | 4.16 | 2.64 | 4.4 | 2.22 | 4.48 | 2.37 | 4.18 | 2.66 | 4.43 | 2.23 | 4.50 | 2.38 | 4.22 | 2.68 | 4.46 | 2.25 | 4.54 | 2.40 | 4.25 | 2.70 | 4.49 | 2.26 | 4.57 | 2.42 | 4.26 | 2.70 | 4.50 | 2.27 | 4.58 | 2.42 |
|  | 0.8 | 4.16 | 2.64 | 4.4 | 2.22 | 4.48 | 2.37 | 4.19 | 2.67 | 4.44 | 2.24 | 4.52 | 2.39 | 4.24 | 2.70 | 4.49 | 2.26 | 4.57 | 2.41 | 4.28 | 2.72 | 4.53 | 2.28 | 4.61 | 2.43 | 4.30 | 2.73 | 4.55 | 2.29 | 4.63 | 2.45 |
|  | 0.9 | 4.15 | 2.64 | 4.39 | 2.21 | 4.47 | 2.36 | 4.20 | 2.67 | 4.44 | 2.24 | 4.52 | 2.39 | 4.25 | 2.70 | 4.50 | 2.27 | 4.58 | 2.42 | 4.29 | 2.73 | 4.54 | 2.29 | 4.62 | 2.44 | 4.32 | 2.75 | 4.57 | 2.31 | 4.65 | 2.46 |
|  | 1 | 4.14,  3.97^a^,  4.10^b^ | 2.63,  2.77^a^,  2.61^b^ | 4.38,  4.38^a^,  4.34^b^ | 2.21,  2.07^a^,  2.19^b^ | 4.46,  4.50^a^,  4.42^b^ | 2.36,  2.33^a^,  2.34^b^ | 4.20 | 2.67 | 4.44 | 2.24 | 4.52 | 2.39 | 4.25,  4.18^b^ | 2.70,  2.66^b^ | 4.50,  4.42^b^ | 2.27,  2.23^b^ | 4.58,  4.50^b^ | 2.42,  2.38^b^ | 4.29 | 2.73 | 4.54 | 2.29 | 4.62 | 2.45 | 4.32,  4.26^b^ | 2.75,  2.71^b^ | 4.58,  4.51^b^ | 2.31,  2.27^b^ | 4.66,  4.59^b^ | 2.46,  2.43^b^ |
| ^a^Ref.^29^, ^b^Ref. ^48^. | | | | | | | | | | | | | | | | | | | | | | | | | | | | | | | |
