# Supplementary material for: Insight into pressure effect on optoelectronic, mechanical, and lattice vibrational properties of nanostructured GaxIn1 − xPySbzAs1 − y − z for the solar cells system
Source: Sci Rep. 2023 Mar 8;13:3891. doi: 10.1038/s41598-023-30681-1 (PMC9995325; doi:10.1038/s41598-023-30681-1)
Supplement: Supplementary file 3 — Supplementary Information 3. [file 41598_2023_30681_MOESM3_ESM.docx]

| **Table 5.** The elastic moduli in (10^11^ Dyn/cm^2^)for the alloy Ga_x_In_1-x_P_y_Sb_z_As_1-y-z_ lattice matched to GaSb for various values of pressure and compositions. | | | | | | | | | | | | | | | | |
| --- | --- | --- | --- | --- | --- | --- | --- | --- | --- | --- | --- | --- | --- | --- | --- | --- |
|  |  | p= 0 kbar | | | p= 30 kbar | | | p= 60 kbar | | | p= 90 kbar | | | p= 120 kbar | | |
| z | x | B_u_ | C_s_ | Y_o_ | B_u_ | C_s_ | Y_o_ | B_u_ | C_s_ | Y_o_ | B_u_ | C_s_ | Y_o_ | B_u_ | C_s_ | Y_o_ |
| 0.2 | 0.0 | 5.44 | 2.49 | 6.47 | 5.83 | 2.66 | 6.93 | 6.25 | 2.85 | 7.42 | 6.64 | 3.03 | 7.90 | 7.01 | 3.20 | 8.34 |
|  | 0.1 | 5.55 | 2.54 | 6.61 | 5.97 | 2.73 | 7.11 | 6.44 | 2.95 | 7.67 | 6.88 | 3.15 | 8.20 | 7.31 | 3.35 | 8.71 |
| 0.4 | 0.0 | 5.2 | 2.37 | 6.17 | 5.46 | 2.48 | 6.46 | 5.68 | 2.57 | 6.70 | 5.85 | 2.64 | 6.89 | 5.86 | 2.64 | 6.89 |
|  | 0.1 | 5.33 | 2.43 | 6.33 | 5.64 | 2.57 | 6.68 | 5.94 | 2.70 | 7.03 | 6.21 | 2.82 | 7.35 | 6.38 | 2.89 | 7.54 |
|  | 0.2 | 5.45 | 2.49 | 6.47 | 5.80 | 2.64 | 6.89 | 6.17 | 2.81 | 7.33 | 6.51 | 2.97 | 7.73 | 6.78 | 3.09 | 8.05 |
|  | 0.3 | 5.55 | 2.54 | 6.61 | 5.94 | 2.72 | 7.07 | 6.37 | 2.91 | 7.57 | 6.75 | 3.08 | 8.03 | 7.08 | 3.24 | 8.43 |
| 0.6 | 0.2 | 5.25 | 2.39 | 6.23 | 5.50 | 2.50 | 6.50 | 5.70 | 2.58 | 6.73 | 5.86 | 2.65 | 6.91 | 5.84 | 2.63 | 6.86 |
|  | 0.3 | 5.38 | 2.45 | 6.39 | 5.68 | 2.58 | 6.73 | 5.96 | 2.71 | 7.06 | 6.21 | 2.82 | 7.35 | 6.33 | 2.87 | 7.48 |
|  | 0.4 | 5.5 | 2.51 | 6.54 | 5.84 | 2.67 | 6.94 | 6.19 | 2.82 | 7.35 | 6.50 | 2.96 | 7.71 | 6.72 | 3.06 | 7.97 |
|  | 0.5 | 5.61 | 2.57 | 6.68 | 5.99 | 2.74 | 7.13 | 6.39 | 2.92 | 7.60 | 6.73 | 3.08 | 8.01 | 7.01 | 3.20 | 8.34 |
| 0.8 | 0.4 | 5.35 | 2.44 | 6.36 | 5.61 | 2.55 | 6.64 | 5.83 | 2.65 | 6.90 | 6.01 | 2.72 | 7.10 | 6.03 | 2.72 | 7.09 |
|  | 0.5 | 5.49 | 2.51 | 6.53 | 5.79 | 2.64 | 6.88 | 6.09 | 2.78 | 7.23 | 6.34 | 2.89 | 7.52 | 6.48 | 2.95 | 7.67 |
|  | 0.6 | 5.61 | 2.57 | 6.68 | 5.97 | 2.73 | 7.10 | 6.32 | 2.89 | 7.52 | 6.62 | 3.02 | 7.87 | 6.84 | 3.12 | 8.13 |
|  | 0.7 | 5.72 | 2.62 | 6.82 | 6.12 | 2.80 | 7.29 | 6.51 | 2.98 | 7.76 | 6.84 | 3.13 | 8.15 | 7.11 | 3.25 | 8.47 |
| 1 | 0.6 | 5.51 | 2.52 | 6.56 | 5.80 | 2.65 | 6.89 | 6.07 | 2.77 | 7.21 | 6.30 | 2.87 | 7.47 | 6.41 | 2.91 | 7.58 |
|  | 0.7 | 5.65 | 2.59 | 6.73 | 5.99 | 2.74 | 7.14 | 6.33 | 2.89 | 7.53 | 6.62 | 3.02 | 7.87 | 6.83 | 3.11 | 8.11 |
|  | 0.8 | 5.77 | 2.65 | 6.89 | 6.17 | 2.83 | 7.35 | 6.54 | 3.00 | 7.80 | 6.87 | 3.15 | 8.19 | 7.15 | 3.27 | 8.51 |
|  | 0.9 | 5.87 | 2.7 | 7.02 | 6.31 | 2.90 | 7.54 | 6.72 | 3.08 | 8.02 | 7.07 | 3.24 | 8.44 | 7.38 | 3.38 | 8.80 |
|  | 1 | 5.96,  5.63^a^ | 2.74,  2.4^c^ | 7.13,  6.99^b^ | 6.43 | 2.96 | 7.69 | 6.86 | 3.15 | 8.20 | 7.22 | 3.32 | 8.63 | 7.54 | 3.46 | 9.01 |
| ^a^Ref.^50^, ^b^Ref. ^43^,^c^Ref. ^29^. | | | | | | | | | | | | | | | | |
