# Supplementary material for: Insight into pressure effect on optoelectronic, mechanical, and lattice vibrational properties of nanostructured GaxIn1 − xPySbzAs1 − y − z for the solar cells system
Source: Sci Rep. 2023 Mar 8;13:3891. doi: 10.1038/s41598-023-30681-1 (PMC9995325; doi:10.1038/s41598-023-30681-1)
Supplement: Supplementary file 4 — Supplementary Information 4. [file 41598_2023_30681_MOESM4_ESM.docx]

| **Table 6.** Calculated bond-stretching (α), bond-bending (β) force constants, and internal strain parameter (ζ) for the alloy Ga_x_In_1-x_P_y_Sb_z_As_1-y-z_ lattice matched to GaSb for various values of pressure and compositions. | | | | | | | | | | | | | | | | | | | | | |
| --- | --- | --- | --- | --- | --- | --- | --- | --- | --- | --- | --- | --- | --- | --- | --- | --- | --- | --- | --- | --- | --- |
|  |  | p= 0 kbar | | | | p= 30 kbar | | | | p= 60 kbar | | | | p= 90 kbar | | | | p= 120 kbar | | | |
| z | x |  | α (N/m) | β (N/m) | ζ |  | α (N/m) | β  (N/m) | ζ |  | α (N/m) | Β  (N/m) | ζ |  | α (N/m) | β (N/m) | ζ |  | α (N/m) | β  (N/m) | ζ |
| 0.2 | 0.0 |  | 30.67 | 7.58 | 0.6 |  | 32.33 | 7.98 | 0.60 |  | 34.18 | 8.44 | 0.60 |  | 35.94 | 8.88 | 0.60 |  | 37.57 | 9.29 | 0.60 |
|  | 0.1 |  | 31.27 | 7.74 | 0.6 |  | 33.12 | 8.19 | 0.60 |  | 35.25 | 8.73 | 0.60 |  | 37.24 | 9.23 | 0.60 |  | 39.16 | 9.72 | 0.60 |
| 0.4 | 0.0 |  | 29.3 | 7.21 | 0.6 |  | 30.31 | 7.44 | 0.61 |  | 31.08 | 7.62 | 0.61 |  | 31.68 | 7.75 | 0.61 |  | 31.48 | 7.67 | 0.61 |
|  | 0.1 |  | 30.01 | 7.4 | 0.6 |  | 31.26 | 7.70 | 0.60 |  | 32.50 | 8.00 | 0.61 |  | 33.61 | 8.26 | 0.61 |  | 34.22 | 8.40 | 0.61 |
|  | 0.2 |  | 30.67 | 7.58 | 0.6 |  | 32.14 | 7.93 | 0.60 |  | 33.76 | 8.33 | 0.60 |  | 35.23 | 8.69 | 0.60 |  | 36.37 | 8.97 | 0.60 |
|  | 0.3 |  | 31.28 | 7.74 | 0.6 |  | 32.95 | 8.15 | 0.60 |  | 34.83 | 8.62 | 0.60 |  | 36.51 | 9.04 | 0.60 |  | 37.97 | 9.40 | 0.60 |
| 0.6 | 0.2 |  | 29.57 | 7.28 | 0.6 |  | 30.48 | 7.49 | 0.61 |  | 31.21 | 7.65 | 0.61 |  | 31.73 | 7.76 | 0.61 |  | 31.37 | 7.64 | 0.61 |
|  | 0.3 |  | 30.3 | 7.48 | 0.6 |  | 31.47 | 7.75 | 0.60 |  | 32.64 | 8.03 | 0.61 |  | 33.61 | 8.26 | 0.61 |  | 33.99 | 8.33 | 0.61 |
|  | 0.4 |  | 30.98 | 7.66 | 0.6 |  | 32.39 | 8.00 | 0.60 |  | 33.89 | 8.36 | 0.60 |  | 35.17 | 8.68 | 0.60 |  | 36.03 | 8.88 | 0.60 |
|  | 0.5 |  | 31.59 | 7.83 | 0.6 |  | 33.22 | 8.22 | 0.60 |  | 34.95 | 8.65 | 0.60 |  | 36.42 | 9.01 | 0.60 |  | 37.58 | 9.29 | 0.60 |
| 0.8 | 0.4 |  | 30.15 | 7.44 | 0.6 |  | 31.09 | 7.65 | 0.61 |  | 31.93 | 7.84 | 0.61 |  | 32.54 | 7.98 | 0.61 |  | 32.34 | 7.90 | 0.61 |
|  | 0.5 |  | 30.9 | 7.64 | 0.6 |  | 32.13 | 7.93 | 0.60 |  | 33.35 | 8.22 | 0.60 |  | 34.35 | 8.46 | 0.60 |  | 34.79 | 8.55 | 0.61 |
|  | 0.6 |  | 31.59 | 7.83 | 0.6 |  | 33.08 | 8.18 | 0.60 |  | 34.58 | 8.55 | 0.60 |  | 35.84 | 8.85 | 0.60 |  | 36.70 | 9.06 | 0.60 |
|  | 0.7 |  | 32.2 | 7.99 | 0.6 |  | 33.91 | 8.41 | 0.60 |  | 35.61 | 8.83 | 0.60 |  | 37.02 | 9.17 | 0.60 |  | 38.13 | 9.44 | 0.60 |
| 1 | 0.6 |  | 31.04 | 7.68 | 0.6 |  | 32.17 | 7.94 | 0.60 |  | 33.25 | 8.19 | 0.60 |  | 34.12 | 8.40 | 0.61 |  | 34.41 | 8.45 | 0.61 |
|  | 0.7 |  | 31.81 | 7.88 | 0.6 |  | 33.23 | 8.22 | 0.60 |  | 34.63 | 8.56 | 0.60 |  | 35.82 | 8.85 | 0.60 |  | 36.62 | 9.04 | 0.60 |
|  | 0.8 |  | 32.49 | 8.07 | 0.6 |  | 34.17 | 8.48 | 0.60 |  | 35.81 | 8.88 | 0.60 |  | 37.19 | 9.22 | 0.60 |  | 38.31 | 9.49 | 0.60 |
|  | 0.9 |  | 33.07 | 8.22 | 0.6 |  | 34.97 | 8.69 | 0.60 |  | 36.76 | 9.13 | 0.60 |  | 38.26 | 9.50 | 0.60 |  | 39.55 | 9.82 | 0.60 |
|  | 1 |  | 33.55  33.16^a^ | 8.35  7.81^b^ | 0.6  0.612^a^ |  | 35.63 | 8.87 | 0.60 |  | 37.51 | 9.33 | 0.60 |  | 39.05 | 9.72 | 0.60 |  | 40.43 | 10.06 | 0.60 |
| ^a^Ref.^53^, ^b^Ref.^54^. | | | | | | | | | | | | | | | | | | | | | |
