# Supplementary material for: Insight into pressure effect on optoelectronic, mechanical, and lattice vibrational properties of nanostructured GaxIn1 − xPySbzAs1 − y − z for the solar cells system
Source: Sci Rep. 2023 Mar 8;13:3891. doi: 10.1038/s41598-023-30681-1 (PMC9995325; doi:10.1038/s41598-023-30681-1)
Supplement: Supplementary file 5 — Supplementary Information 5. [file 41598_2023_30681_MOESM5_ESM.docx]

| **Table 7.** Transverse effective charge$\left( e_{T}^{*} \right),$ the phonon frequencies ω_LO_ and ω_TO_ (10^13^ s^-1^) for the alloy Ga_x_In_1-x_P_y_Sb_z_As_1-y-z_ lattice matched to GaSb for various values of pressure and compositions. | | | | | | | | | | | | | | | | | | | | | | | | | | |
| --- | --- | --- | --- | --- | --- | --- | --- | --- | --- | --- | --- | --- | --- | --- | --- | --- | --- | --- | --- | --- | --- | --- | --- | --- | --- | --- |
|  |  | p= 0 kbar | | | | | p= 30 kbar | | | | | p= 60 kbar | | | | | p= 90 kbar | | | | | p= 120 kbar | | | | |
| z | x | $\left( e_{T}^{*} \right)$ | ω_LO_ | ω_TO_ |  |  | $\left( e_{T}^{*} \right)$ | ω_LO_ | ω_TO_ |  |  | $\left( e_{T}^{*} \right)$ | ω_LO_ | ω_TO_ |  |  | $\left( e_{T}^{*} \right)$ | ω_LO_ | ω_TO_ |  |  | $\left( e_{T}^{*} \right)$ | ω_LO_ | ω_TO_ |  |  |
| 0.2 | 0.0 | 2.1 | 4.5 | 4.22 |  |  | 2.99 | 6.91 | 6.45 |  |  | 2.12 | 5.46 | 5.11 |  |  | 2.10 | 6.07 | 5.70 |  |  | 2.07 | 6.69 | 6.31 |  |  |
|  | 0.1 | 2.03 | 4.38 | 4.14 |  |  | 2.96 | 6.96 | 6.57 |  |  | 2.01 | 5.48 | 5.20 |  |  | 1.97 | 6.19 | 5.91 |  |  | 1.92 | 6.98 | 6.71 |  |  |
| 0.4 | 0.0 | 2.22 | 5.08 | 4.67 |  |  | 2.29 | 5.43 | 4.92 |  |  | 2.35 | 5.79 | 5.19 |  |  | 2.39 | 6.16 | 5.47 |  |  | 2.46 | 6.42 | 5.60 |  |  |
|  | 0.1 | 2.16 | 4.89 | 4.54 |  |  | 2.22 | 5.24 | 4.81 |  |  | 2.25 | 5.66 | 5.17 |  |  | 2.27 | 6.10 | 5.56 |  |  | 2.32 | 6.46 | 5.85 |  |  |
|  | 0.2 | 2.1 | 4.73 | 4.44 |  |  | 2.15 | 5.11 | 4.75 |  |  | 2.15 | 5.60 | 5.21 |  |  | 2.16 | 6.11 | 5.70 |  |  | 2.17 | 6.57 | 6.11 |  |  |
|  | 0.3 | 2.03 | 4.6 | 4.35 |  |  | 2.07 | 5.01 | 4.72 |  |  | 2.06 | 5.59 | 5.28 |  |  | 2.04 | 6.18 | 5.85 |  |  | 2.04 | 6.73 | 6.38 |  |  |
| 0.6 | 0.2 | 2.2 | 5.39 | 4.97 |  |  | 2.28 | 5.69 | 5.17 |  |  | 2.34 | 6.02 | 5.42 |  |  | 2.39 | 6.38 | 5.67 |  |  | 2.47 | 6.61 | 5.76 |  |  |
|  | 0.3 | 2.13 | 5.2 | 4.85 |  |  | 2.20 | 5.52 | 5.09 |  |  | 2.24 | 5.92 | 5.44 |  |  | 2.27 | 6.34 | 5.79 |  |  | 2.33 | 6.66 | 6.02 |  |  |
|  | 0.4 | 2.06 | 5.05 | 4.75 |  |  | 2.12 | 5.40 | 5.05 |  |  | 2.14 | 5.89 | 5.49 |  |  | 2.16 | 6.37 | 5.93 |  |  | 2.20 | 6.76 | 6.27 |  |  |
|  | 0.5 | 1.99 | 4.91 | 4.66 |  |  | 2.04 | 5.32 | 5.03 |  |  | 2.04 | 5.89 | 5.57 |  |  | 2.05 | 6.43 | 6.08 |  |  | 2.07 | 6.89 | 6.50 |  |  |
| 0.8 | 0.4 | 2.15 | 5.76 | 5.37 |  |  | 2.23 | 6.03 | 5.54 |  |  | 2.29 | 6.37 | 5.80 |  |  | 2.34 | 6.74 | 6.07 |  |  | 2.42 | 6.99 | 6.19 |  |  |
|  | 0.5 | 2.07 | 5.61 | 5.28 |  |  | 2.15 | 5.91 | 5.51 |  |  | 2.19 | 6.33 | 5.87 |  |  | 2.22 | 6.76 | 6.23 |  |  | 2.28 | 7.09 | 6.48 |  |  |
|  | 0.6 | 1.99 | 5.48 | 5.22 |  |  | 2.06 | 5.84 | 5.52 |  |  | 2.08 | 6.34 | 5.98 |  |  | 2.11 | 6.83 | 6.42 |  |  | 2.15 | 7.23 | 6.76 |  |  |
|  | 0.7 | 1.92 | 5.37 | 5.16 |  |  | 1.96 | 5.81 | 5.55 |  |  | 1.97 | 6.40 | 6.11 |  |  | 1.99 | 6.94 | 6.62 |  |  | 2.02 | 7.40 | 7.03 |  |  |
| 1 | 0.6 | 2.06 | 6.3 | 5.95 |  |  | 2.14 | 6.54 | 6.11 |  |  | 2.20 | 6.91 | 6.40 |  |  | 2.24 | 7.32 | 6.74 |  |  | 2.31 | 7.64 | 6.96 |  |  |
|  | 0.7 | 1.97 | 6.22 | 5.94 |  |  | 2.04 | 6.52 | 6.17 |  |  | 2.08 | 6.97 | 6.58 |  |  | 2.11 | 7.45 | 7.00 |  |  | 2.15 | 7.85 | 7.34 |  |  |
|  | 0.8 | 1.87 | 6.19 | 5.97 |  |  | 1.93 | 6.56 | 6.29 |  |  | 1.95 | 7.11 | 6.81 |  |  | 1.97 | 7.64 | 7.31 |  |  | 2.01 | 8.12 | 7.75 |  |  |
|  | 0.9 | 1.78 | 6.18 | 6.01 |  |  | 1.82 | 6.67 | 6.46 |  |  | 1.83 | 7.32 | 7.09 |  |  | 1.85 | 7.90 | 7.65 |  |  | 1.87 | 8.45 | 8.17 |  |  |
|  | 1 | 1.69,  1.91^b^,  1.89^c^ | 6.18,  5.65^a^ | 6.04,  5.48^a^ |  |  | 1.72 | 6.83  ,5.89^a^ | 6.67  ,5.66^a^ |  |  | 1.72 | 7.59  ,6.54^a^ | 7.41  ,6.30^a^ |  |  | 1.74 | 8.20  ,7.21^a^ | 8.01  ,6.94^a^ |  |  | 1.75 | 8.81  ,7.85^a^ | 8.60  ,7.57^a^ |  |  |
| ^a^Ref.^48^, ^b^Ref. ^41^, ^c^Ref.^22^. | | | | | | | | | | | | | | | | | | | | | | | | | | |
